# Accuracy and consistency of weights provided by home bathroom scales

**DOI:** 10.1186/1471-2458-13-1194

**Published:** 2013-12-17

**Authors:** Meredith Yorkin, Kim Spaccarotella, Jennifer Martin-Biggers, Virginia Quick, Carol Byrd-Bredbenner

**Affiliations:** 1 Department of Nutritional Sciences, Rutgers, The State University of New Jersey, 26 Nichol Avenue, Davison Hall, 08901, New Brunswick, NJ, USA; 2 Division of Intramural Population Health Research, Eunice Kennedy Shriver National Institute of Child Health & Human Development, National Institutes of Health, 6100 Executive Boulevard, 20892, Bethesda, MD, USA

**Keywords:** Body weight, Body mass index, Validity

## Abstract

**Background:**

Self-reported body weight is often used for calculation of Body Mass Index because it is easy to collect. Little is known about sources of error introduced by using bathroom scales to measure weight at home. The objective of this study was to evaluate the accuracy and consistency of digital versus dial-type bathroom scales commonly used for self-reported weight.

**Methods:**

Participants brought functioning bathroom scales (n = 18 dial-type, n = 43 digital-type) to a central location. Trained researchers assessed accuracy and consistency using certified calibration weights at 10 kg, 25 kg, 50 kg, 75 kg, 100 kg, and 110 kg. Data also were collected on frequency of calibration, age and floor surface beneath the scale.

**Results:**

All participants reported using their scale on hard surface flooring. Before calibration, all digital scales displayed 0, but dial scales displayed a mean absolute initial weight of 0.95 (1.9 SD) kg. Digital scales accurately weighed test loads whereas dial-type scale weights differed significantly (p < 0.05). Imprecision of dial scales was significantly greater than that of digital scales at all weights (p < 0.05). Accuracy and precision did not vary by scale age.

**Conclusions:**

Digital home bathroom scales provide sufficiently accurate and consistent weights for public health research. Reminders to zero scales before each use may further improve accuracy of self-reported weight.

## Background

Self-reported heights and weights are often used in public health research with adults, children and families because these data are easy and inexpensive to collect [1]. However, self-reported and expert-measured weights may differ by factors such as age, sex, and perceived weight status [2-4]. In addition to parents reporting their own weight, they also may be asked to report the weight of their children. A child’s weight status as well as parental perception of their child’s weight affect accuracy of reports [4,5]. A recent study reported that parents of overweight children ages 2 to 6 years old erroneously overestimated their child’s weight, but parents of older overweight children and adolescents underestimated their child’s weight [4]. Similar findings were reported using data from two nationally representative surveys [6]. Others have noted mothers underestimate child weight [7,8].

Inaccuracies in reported weights often are attributed to social desirability and/or erroneous measurements or recalls [1,9,10]. Minimal research, however, has examined the accuracy of a common tool used to measure self-reported weights, that is, home bathroom scales. The limited available data suggest that home scales, as well as medical grade scales used by physicians, can vary in accuracy and precision [11]. A study of 37 dial-type bathroom scales in British clinics reported inaccuracies of more than 1% compared with a calibrated electronic scale, suggesting that digital scales may be more accurate [12]. Further, an evaluation of 233 scales (type not specified) from United States primary care, diabetology and endocrinology clinics, and fitness and weight loss centers found that more than a quarter of the scales were more than 0.9 kg imprecise when tested with a 45.5 kg standard weight. At 113.6 kg, about one in five scales was imprecise by more than 2.7 kg, or about 1 Body Mass Index (BMI) unit [10]. Several factors, such as type of flooring, foot placement on the scale, and type of clothing or shoes worn during weighing, may influence accuracy of scales [13].

Widespread implementation of community-based obesity prevention programs targeting children and families is currently underway [14-16]. Some of these programs, and the research used to develop them, rely on self-reported weight or BMI, which is calculated using weight and reported as a primary outcome or measure of intervention effectiveness [14,17,18]. A major limitation to establishing intervention (in)effectiveness is inaccurate weight reports [1,19,20]. To advance the work of public health professionals in implementing effective programming aimed at ameliorating the obesity epidemic, it is important to increase the accuracy of self-reports. Thus, the goal of this study was to assess the accuracy of home bathroom scales to better understand how their use in the common process of self-weighing may affect accuracy of self-reported weight data [21].

## Methods

### Participants and procedure

Notices were posted to recruit study participants from the university campus. Participants included faculty, staff, students and parents of children attending a preschool run by the university. The Rutgers University Institutional Review Board approved the procedures, and all participants gave informed consent.

Participants brought their functioning home bathroom scale to a central location for assessment and completed a questionnaire describing the scale’s age, type of flooring in the location where the scale is used in the home, frequency of use, and calibration history. Trained researchers recorded scale condition (i.e., new, light wear, heavily worn or outward evidence of damage), type (i.e., dial or digital), units and increments of measurement, maximum capacity, and weight displayed upon arrival at the testing site.

The procedure for evaluating scale accuracy was modeled on previous research [10]. The accuracy of scales in measuring weight load was assessed using National Institute of Standards and Technology (NIST) Class F calibration weights at the following test loads: 10 kg, 25 kg, 50 kg, 75 kg, 100 kg, and 110 kg. To determine accuracy in measuring weight distribution (i.e., distributed over a human body or concentrated in a calibration weight), two humans were weighed in addition to the calibration weights (i.e., one researcher and a second researcher holding a 10 kg calibration weight close to the body between the waist and hips). To determine consistency in weight measurements, all weight assessments were measured in duplicate (Round 1 and Round 2). During Round 1, each scale was used to assess 8 loads: the two researchers and the 6 calibration weights. Round 2 was the same as Round 1 and was conducted immediately after Round 1. At the end of each Round 2, a calibrated research scale was used to weigh in duplicate the first researcher and the second researcher holding a 10 kg calibration weight close to the body between the waist and hips. The calibrated research scale weights were used as the “standard” for comparing the two researcher weights registered by the home scale. The NIST calibration weights served as their own comparison to those registered by the home scale. Prior to Round 1 and in between each test load as necessary, all scales were calibrated to register zero when no weight was applied. Data were recorded in real time using a computerized spreadsheet.

### Statistical analysis

Data were analyzed using SPSS version 21.0 (Chicago, IL). Paired samples *t*-tests were used to test for differences in scale consistency between Round 1 and 2. One group t-tests were used to estimate differences between the displayed weight and actual weights of the calibration weights. Unpaired *t*-tests were used to estimate the precision between different types of scales, and multinomial regression was used to examine the percent of digital versus dial scales with various degrees of weight imprecision. Absolute weights were used to avoid the possibility of underweight errors canceling out overweight errors. Differences were considered significant at p < 0.05. Values are reported as means and standard deviations (SD) unless otherwise noted.

## Results

Of the 67 bathroom scales that were received, 6 scales were eliminated due to damage or improper functioning (i.e. did not register a value when tested with the calibration weights) or because they were not a home scale. Of the final sample (n = 61), 18 were dial (30%) and 43 (70%) were digital scales from 16 various manufacturers. All were new, like new, or had light wear. The bathroom scales’ precision increments were 0.045 kg, 0.091 kg., 0.15 kg, and 0.45 kg (21%, 38%, 13%, and 28%, respectively). The maximum weight capacities ranged from 123 kg to 181 kg, with the most common capacity being 136 kg (35%). Dial scales were significantly older (p = 0.042; mean age 6.0 (6.9 SD) years old) than digital scales (mean age 3.6 (2.3 SD) years old). When the scales were first observed, all digital scales displayed 0, whereas dial scales displayed a mean absolute weight of 0.95 (1.9 SD) kg, with a range of -0.45 to 7.9 kg.

All participants reported using their bathroom scale on hard flooring. Scales were used daily (21%), weekly (46%), or monthly (27%), and 5% used their scale yearly or less than once a year. Participants reported infrequently calibrating their scale; only 28% calibrated the scale each time or most of the time before using it. Of these participants, 64% had dial-type scales.

Mean weight between Rounds 1 and 2 differed significantly only for dial scales tested with a 75 kg calibration weight (p = 0.028). For all other test loads, scales consistently registered the same weight, regardless of the scale type and load. Per 45.4 kg, the difference in absolute weight registered between Round 1 and 2 averaged approximately 0.11 kg (range from 0.0 kg to 0.6 kg), with the largest errors for the 10 kg calibration weight.

**Figure 1:**
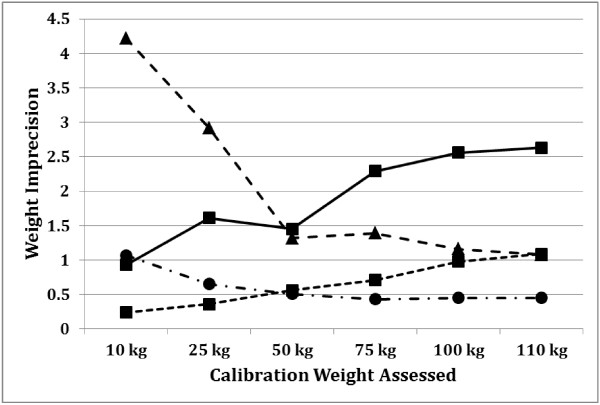
Home bathroom scale weight imprecision at 6 calibration weight test loads*. *Dial n = 18, Digital 10 kg *n* = 37; 25 kg *n* = 42; 50, 75, 100 kg *n* = 43; 110 kg *n* = 41. Triangle marker and dashed line: Dial% of Weight (n = 18). Circle marker and dashed line: Digital% of Weight (n = 35). Square marker and solid line: Dial (n = 18). Square marker and dashed line: Digital (n = 35).

Table 1 compares accuracy of the mean weight registered by the home scales to the known calibration weight tested. For dial scales only, significant differences of weights between the test load and the weight displayed on the home scales occurred for all calibration weights and both weighed researchers (p < 0.05 for all). In contrast, significant differences for digital scale accuracy occurred only with the 75 kg calibration weight (p < 0.05). Dial scales were significantly more imprecise than digital scales at all test weights (Table 2). Figure 1 visually displays the absolute weight imprecision across each calibration weight test load. Scales became increasingly less precise as weight load increased; however, imprecision as a percent of total weight test load was inversely related to weight load. Accuracy did not vary by age of the scale. There was no significant difference between dial scales ≤3 years old (n = 10) and those ≥3 years old (n = 8), and no differences between digital scales ≤3 years old (n = 25) compared to those ≥3 years old n = 18). Thus, scale type, not age, is likely the source of inaccuracy and imprecision.

**Table 1:** Scale accuracy: mean weights registered by scales compared to calibration weight tested

***Calibration weight***	***n***	***Mean***	***SD***	
***Scale***		***(kg)***	***(kg)***	P*
**10 kg**				
All scales	55	9.8	0.3	0.007
Dial	18	9.5	0.4	<0.001
Digital	37	9.9	0.2	>0.05
**25 kg**				
All scales	60	24.5	0.5	0.002
Dial	18	24.0	0.5	<0.001
Digital	42	24.8	0.4	>0.05
**50 kg**				
All scales	61	49.4	0.6	>0.05
Dial	18	49.1	0.7	0.013
Digital	43	49.6	0.5	>0.05
**75 kg**				
All scales	61	74.1	0.8	0.084
Dial	18	73.2	0.7	<0.001
Digital	43	74.4	0.5	0.037
**100 kg**				
All scales	61	98.1	6.4	>0.05
Dial	18	97.2	0.9	0.001
Digital	43	98.1	7.6	>0.05
**110 kg**				
All scales	59	108.7	1.1	>0.05
Dial	18	107.7	1.0	0.001
Digital	41	109.2	0.8	>0.05
**Weight of researcher 1**				
(Mean weight = 70.1 kg research scaleused as accuracy standard)				
All scales	61	69.2	9.2	0.005#
Dial	18	70.3	10.1	<0.001#
Digital	43	68.8	8.8	>0.05#
**Weight of researcher 2 holding 10 kg weight close to his/her body** (Mean weight = 71.0 kg on research scale used as accuracy standard)				
All scales	61	70.2	9.5	0.020#
Dial	18	70.7	0.2	<0.001#
Digital (n = 43)	43	70.1	9.3	>0.05#

SD = standard deviation, CW = calibration weight.

*One sample t-test (calibration weight compared to weight registered on scale; except for researchers’ weights).

#Paired t-tests (researcher weight on research scale compared to researcher weight registered on scale).

**Table 2:** Comparison of dial and digital scale absolute value of weight imprecision at various test loads

***Calibration weight***	***n***	**Mean**	**SD**	*P
***Scale***		**(kg)**	**(kg)**	
**10 kg**				
All scales	55	0.2	0.3	
Dial	18	0.4	0.3	<0.001
Digital	37	0.1	0.2	
**25 kg**				
All scales	60	0.4	0.5	
Dial	18	0.7	0.5	<0.001
Digital	42	0.2	0.3	
**50 kg**				
All scales	61	0.4	0.4	
Dial	18	0.7	0.5	0.004
Digital	43	0.3	0.3	
**75 kg**				
All scales	61	0.6	0.6	
Dial	18	1.0	0.7	<0.001
Digital	43	0.4	0.4	
**100 kg**				
All scales	60	0.7	0.7	
Dial	18	1.2	0.9	0.001
Digital	42	0.54	0.5	
**110 kg**				
All scales	59	0.8	0.8	
Dial	18	1.2	1.0	0.007
Digital	41	0.6	0.6	

SD = standard deviation, CW = calibration weight.

*Data are from unpaired *t*-tests comparing the absolute values of imprecision at each test load registered by dial versus digital HBS.

Multinomial regression was used to examine associations between absolute weight imprecision (i.e., <0.45 kg., 0.45 to <0.91 kg., 0.91 to <1.8 kg., 1.8 to < 2.7 kg, and ≥2.7 kg.) and type of scale. The majority of scales were precise within 0.9 kg of the actual weight of the load tested, but the extent of imprecision increased as the tested weight increased. For instance, at 50 kg, less than 2% of all tested scales were off by at least 1.8 kg; whereas the proportion rose to nearly 14% when the 110 kg calibration weight was tested. Further, the overall imprecision of dial scales was significantly greater than that of digital scales at all weights, with nearly 17% having a precision error of at least 2.7 kg or 1 BMI unit at a test load of 99.8 kg or greater (p < 0.05).

## Discussion

Findings from this study indicate that home bathroom scales are consistent in the weights measured. Dial scales were significantly more imprecise than digital scales at all calibration weight test loads measured with digital home scale weights differing significantly at the 75 kg test load. The imprecision at the 75 kg test load likely is due to human error in recording of data (e.g. incorrectly recording the weight as measured by the scale) during scale testing. The finding that scale precision was significantly higher at all test loads for digital versus dial-type scales confirms previous reports of significant, positive correlations between scale precision and accuracy [10]. Although the reasons for the differences between dial and digital scale precision are not completely clear, digital scales have fewer moving parts to get out of alignment or become damaged and have mechanisms to automatically set the starting weight to zero [9].

Although dial scales were significantly more imprecise than digital scales, absolute imprecision tended to be within 0.91 kg of calibration weights, a level far below the threshold that would cause weight to result in an error of one BMI unit (i.e., approximately 2.7 kg) [22]. It is also within the ~0.5 kg fluctuation in body weight considered as normal daily variation in healthy adults [22,23]. For children, expected daily variation in weight is about 1.5 ± 0.5% of their body weight or about 375 to 750 g for children who weigh 25 to 50 kg [20]. For very young children, a difference of 0.91 kg could place them in a different BMI-for-age percentile, potentially resulting in misclassification of their BMI [22]. The present study suggests that dial scales in particular lack accuracy at weights below 10 kg; thus, use of a dial scale to weigh small children should be avoided. Given that weight distribution did not affect scale accuracy, it may be feasible for parents with young children who have dial scales to derive child weight by subtracting their own weight from their weight while holding the child. Future research is needed to explore the accuracy and feasibility of this technique.

This study suggests that inaccuracies in self-reported weight likely are due in large part to human bias and/or reporting or recall errors and not the home bathroom scales. Other studies have found that mothers with less education are more likely to provide inaccurate self-reported weight [5]. The majority of participants had a digital scale, suggesting that these are more likely to be used in self-reported weight. In addition, the finding that all scales were used on hard flooring, such as tile, wood, or cement, indicates that consumers understand the effect of floor coverings on scale accuracy, or scales are used in bathrooms, which coincidentally have hard flooring. The infrequent calibration history, however, suggests that consumers could benefit from reminders to set scales to zero before taking weights. Alternately, self-zeroing digital scales can help overcome this potential source of measurement error.

## Conclusions

These findings suggest that errors made in self-reported weights are more likely due to human error or social desirability than scale inaccuracy. Importantly, this study suggests that home bathroom scales, especially digital scales, provide sufficiently accurate and consistent results for use in public health research. Providing participants with instructions for calibrating their home bathroom scales before use may further improve the accuracy of self-reported weight. In addition, researchers can query participants about scale characteristics such as type of flooring, room in which scale is housed, presence of moisture or steam in the environment that may cause rusting (i.e. in a bathroom), participant ability to calibrate the scale, age of scale and signs of wear to aid in interpretation of the data collected. Future research should also develop and validate instructions for measuring height at home to improve the overall accuracy of self-reported BMI. Finally, researchers should determine whether other factors, such as socioeconomic status or participant age, affect the type of scale owned, the amount of wear, and the frequency with which it is calibrated.

## Competing interest

The authors declare that they have no competing interests.

## Authors’ contributions

The following co-authors contributed to the work: MY in data collection, manuscript preparation and manuscript review. KS in manuscript preparation and manuscript review. VQ in data analysis, manuscript preparation and manuscript review. JMB in data collection and manuscript review. CBB in study design and manuscript review. All authors read and approved the final manuscript.

## Pre-publication history

The pre-publication history for this paper can be accessed here:

http://www.biomedcentral.com/1471-2458/13/1194/prepub
